# Molecular Identification and Phylogenetic Classification of *Leishmania* spp. Isolated from Human Cutaneous Leishmaniasis in Iran: A Cross-sectional Study

**Published:** 2018

**Authors:** Anita MOHAMMADIHA, Abdolhossein DALIMI, Mehdi MOHEBALI, Iraj SHARIFI, Mohammadreza MAHMOUDI, Asad MIRZAEI, Adel SPOTIN, Mahmoodreza BEHRAVAN, Mehdi KARIMI, Mohsen ARBABI, Shahram NEKOEIAN, Reza KALANTARI, Behzad GHORBANZADEH

**Affiliations:** 1. Dept. of Parasitology, Faculty of Medical Sciences, Tarbiat Modares University, Tehran, Iran; 2. Dept. of Medical Parasitology and Mycology, School of Public Health, Tehran University of Medical Sciences, Tehran, Iran; 3. Center for Research of Endemic Parasites of Iran (CREPI), Tehran University of Medical Sciences, Tehran, Iran; 4. Leishmaniasis Research Center, Dept. of Parasitology, Faculty of Medicine, Kerman University of Medical Sciences, Kerman, Iran; 5. Dept. of Microbiology and Parasitology, Faculty of Medicine, Guilan University of Medical Sciences, Rasht, Iran; 6. Dept. of Parasitology, Faculty of Medicine, Ilam University of Medical Sciences, Ilam, Iran; 7. Immunology Research Center, Tabriz University of Medical Sciences, Tabriz, Iran; 8. Esfarayen Faculty of Medical Sciences, Esfarayen, Iran; 9. Infectious Diseases Research Center, Microbiology Dept., Birjand University of Medical Sciences, Birjand, Iran; 10. Dept. of Parasitology and Mycology, School of Medicine, Kashan University of Medical Sciences, Kashan, Iran; 11. Dept. of Cellular and Molecular Biology, School of Medicine, Isfahan University of Medical Sciences, Isfahan, Iran

**Keywords:** Phylogeny, *L. major*, *L. tropica*, PCR-RFLP, Human, Iran

## Abstract

**Background:**

In Iran, both forms of cutaneous (CL) and visceral leishmaniasis (VL) have been reported; so the accurate species identification of the parasite(s) and the analysis of genetic diversity are necessary.

**Methods:**

The smears were collected from lesions samples of 654 patients with CL, who attended local health centers in 12 provinces of Iran during 2013–2015. The smears were checked for the presence of amastigotes by light microscopy. DNA of 648 *Leishmania* isolates, amplified by targeting a partial sequence of ITS (18S rRNA–ITS1–5.8S rRNA–ITS2) gene. Twenty-five of all the amplicons were sequenced and analyzed with restriction fragment length polymorphism (RFLP) using the *Taq*1 enzyme.

**Results:**

All the smears were positive microscopically. The PCR-RFLP analysis revealed that 176 (27%) CL patients were infected with *L. tropica* and, 478 (73%) with *L. major*. The dominant species in all over Iran is *L. major*. The sequencing results of all CL patients and RFLP analysis confirmed each other. Based on our phylogenetic tree, 25 ITS DNA sequences were grouped into two clusters representing *L. major* and *L. tropica* species. Phylogenetic tree derived from the ITS sequences supports a clear divergence between *L. major* from the other species.

**Conclusion:**

Discrimination of Iranian Leishmania isolates using ITS gene gives us this opportunity to detect, identify, and construct the phylogenetic relationship of Iranian isolates.

## Introduction

The *Leishmania* parasites are responsible for a range of infectious diseases widespread in the Old and New World with great epidemiological diversity. Cutaneous Leishmaniasis (CL) is known as one of the most important chronic cutaneous ulcerative lesions with four clinical forms; Acute cutaneous, Chronic cutaneous, Lupoid and Diffuse cutaneous.

In Iran, two forms of Cutaneous Leishmaniasis (CL) exist; zoonotic cutaneous leishmaniasis (ZCL) caused by *L. major* which occurs mostly in rural areas (1,2) and anthroponotic cutaneous leishmaniasis (ACL) caused by *L. tropica* mainly in large cities (3). CL distributes in some geographical locations such as the northeast (4), center (5), west (6), east and south (7–9) of Iran. Annually, about 20000 cases of CL are reported from different parts of Iran, but the actual amount is several times higher (10, 11). CL is endemic in 15 out of 31 provinces and in the recent years, favorable climatic and ecological conditions for its vectors and reservoirs been created the active foci in some areas (12).

Epidemiological studies, taxonomic and population genetics investigations, the prognosis of the disease, assessing a specific chemotherapeutic regimen, effective control of the disease and avoiding the disease transmission; all, are essential for a control program in endemic areas such as in Iran. Therefore, the accurate identification of the parasite(s) and the analysis of genetic diversity of the parasites need to be felt (13, 14).

PCR-based assays routinely constitute the main molecular diagnostic technique of discrimination of *Leishmania* parasites in any kind of infected tissues. Different techniques which harbor PCR-assays, different genetical targets, and various post-PCR techniques enable researchers to conduct a wide range of investigations with various gains, all over the world (15–17). The majority of *Leishmania* cells contain only hundreds of tandemly repeated nuclear ribosomal genes (rDNA), which provide species-specific sequence markers most frequently detected by Restriction Fragment Length Polymorphism (RFLP) analysis of one-step PCR products. In recent years, few studies have carried out to reveal the phylogenetic relationship between Iranian isolates in Iran by different methods, various geographical regions for sampling and sample sizes on reservoir or final hosts (18, 19).

We have used a conventional PCR that amplifies the wide region of ITS gene; including 18S rRNA partially, ITS1, 5.8S rRNA completely and ITS2 partially for identification followed by sequencing and RFLP, constructing a phylogenetic tree and analyzing of a number of cutaneous host-infecting *Leishmania* isolates from different endemic areas of Iran.

## Materials and Methods

**Fig. 1:**
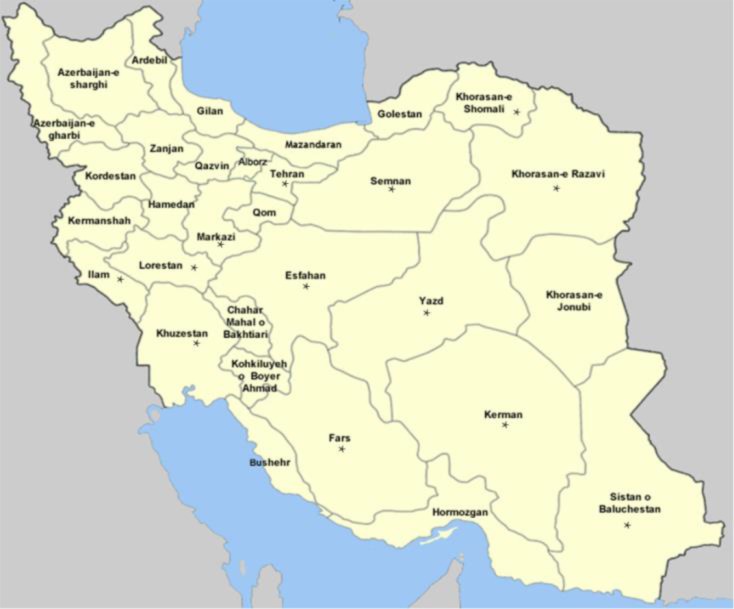
The samples were collected from 12 provinces; Khorasan-Razavi, North-Khorasan, Sistan-Baluchistan, Yazd, Isfahan, Ilam, Lorestan, Fars, Kerman, Khuzestan, Semnan and Tehran provinces which indicated with (^*^) in the map

This study was carried out in a widespread descriptive-cross-sectional manner from 12 provinces of Iran (Khorasan-Razavi 96 samples; North-Khorasan 96 samples), in the south-east (Sistan-Baluchistan 17 samples), in the center (Yazd 15 samples; Isfahan 114 samples), in the west (Ilam 73 samples; Lorestan 12 samples), in the south (Fars 81 samples; Kerman 37 samples), in the southwest (Khuzestan 42 samples), in the north (Semnan 11 samples; Tehran 54 samples) during the summer of 2013 until summer of 2015. Totally, 648 isolates were collected from patients with CL. (Fig. 1 and Table 1).

**Table 1:** List of 648 Iranian isolates of *Leishmania* spp. characterized by ITS-PCR-RFLP including the 4 accession numbers of the sequenced PCR products. Isolated were from cutaneous cases of leishmaniasis of Iran (2013–2015)

***No***	***Province***	***Sample No.***	***Cities***	***Species***	Accession Numer In GenBank^**^
1	Ilam	73	Dehloran/Mehran	*L.tropica*	
				*L.major*	
2	Kerman	37	Kerman/Bam	*L.tropica*	KR706374/Kerman, tro
3	Fars	81	Shiraz/Neyriz/Fasa	*L.tropica*	
				*L.major*	
4	Semnan	11	Damghan	*L.major*	
5	Khorasan-Razavi	96	Mashhad/Sarakhss	*L.tropica*	KP893242/Mashhad, tro
				*L.major*	KP874100/Mashhad, maj
6	Yazd	15	Yazd	*L.tropica*	
				*L.major*	KR868689/Yazd, maj
7	Tehran	54	Ray/Varamin	*L.tropica*	
				*L.major*	
8	Khorasan-North	96	Esfarayen/Jajarm	*L.tropica*	
				*L.major*	
9	Isfahan	114	Isfahan/Kashan	L.tropica^*^	
				*L.major*	
10	Khuzestan	42	Shoosh/Soosangerd/Omidiyeh	*L.tropica*	
				*L.major*	
11	Sistan & Baloochestan	17	Saravan/Zabol	L.tropica^*^	
				*L.major*	
12	Lorestan	12	Khorram-abad	*L.major*	

* Not involved in phylogenic tree construction.

** The accession numbers of *Leishmania* ITS sequences in Iranian species submitted in GenBank

Skin lesions samples were collected from 648 CL patients from 12 endemic foci of CL in Iran. The questionnaires were completed for each individual; age, sex of patients and type, number and location of CL lesions, and furthermore, the history of diseases in affected areas. The characteristics and geographical origins of the human cutaneous isolates are listed in Table 1. Cutaneous samples (smears) were passively taken from acute skin lesions in local medical health centers.

After sterilizing around the lesions/nodules with absolute ethanol, a small incision was made in the margin of the lesion using a disposable lancet and some tissue and exudates were removed by scraping, finally smeared on glass slides.

**Samples:** The scraping smears from the lesions were air-dried, fixed in methanol, stained with Giemsa 10%, and examined for the presence of the amastigotes by light microscopy. Microscope slides were examined under high magnification (1000×) for the presence of *Leishmania* amastigotes. The amastigotes were quantitated as follows: grade 0=no amastigotes per 1000 high-power field; 1=1– 10 amastigotes per 1000 high-power fields; 2=1–10 amastigotes per 100 high-power fields; 3=1–10 amastigotes per 10 high-power fields; 4=1–10 amastigotes per high-power field; 5=10–100 amastigotes per high-power field; 6=>100 amastigotes per high-power field (20, 21).

**Negative and positive control:** Negative controls (NC, samples with no DNA of *Leishmania*) and Positive controls (PC) were prepared from the Leishmaniasis Laboratory at the School of Public Health (SPH), TUMS. All PC, NC and clinical samples were applied for PCR in the same condition.

### Culture of Reference Strains of Leishmania

Reference strains of three old world species of the subgenus *Leishmania* were used: *L. major* (MRHO/IR/75/ER), *Leishmania tropica* (MHOM/IR/99/YAZ1) and *L. infantum* (MCAN/IR/07/Moheb-gh). They were stored in liquid nitrogen and when necessary, culture was carried out in biphasic culture media (prepared from nutrient agar containing 10% whole rabbit blood overlaid with liver infusion tryptose broth containing 100–200 UI/ml penicillin G and 1 μg/ml streptomycin with 10%–20% heat-inactivated fetal bovine serum (Atlanta Biological, Atlanta, CA). The cultures were incubated at 21 °C for up to six weeks and examined weekly for the presence of promastigotes. Meanwhile, RPMI1640 (GIBCO) media used for mass production of promastigotes.

### DNA Isolation

**Giemsa-stained slides:** First of all, slides discolored by incubating in absolute ethanol for 10 min, dried at room temperature, then were covered by 1 mL sterile distilled water and incubated for 10 min at room temperature. The smears removed completely and transferred to 1.5 ml reaction tube, centrifuged at 8000×g for 5 min. Finally, the supernatants discarded and the pellets were ready for DNA extraction.

**DNA extraction:** DNA was extracted with the DNG-plus Extraction Kit (Cinnagen, Iran) according to the manufacturer′s instructions. The DNA pellet was dissolved in 50 μL of sterile distilled water and incubated in a water bath at 65 °C for 5 min. DNA concentration and quality were determined using Nanodrop ND-1000 Spectrophotometer (Nanodrop Technologies, Wilmington, DE, USA) at 260 and 280 nm. DNA samples with A260/A280 ratios between 1.8 and 2 were selected and stored at −20 °C for further analysis.

### PCR Amplification by ITS-primers

Primers were designed based on the ITS region, including forward primer MOF (5′-GCAGCTGGATCATTTTCCGATG-3′) and reverse primer MOR (5′-GAATTCAACTTCGCGTTGGCC-3′). The PCR product size stays between 800 and 860 bp, based on *Leishmania* spp.

### RFLP Analysis of Amplified ITS gene.

Restriction fragment length polymorphism (RFLP) analysis of the ITS fragments was performed on the ITS amplicons, obtained from 654 smear samples, and 3 the reference strains, using the restriction enzyme *Taq1* (1 μL) (Promega, USA) without prior purification. The restriction fragments obtained were compared with the molecular profiles of the WHO reference strains of *L. tropica* (MHOM/IR/99/YAZ1), and *L. major* (MRHO/IR/75/ER) *L. Infantum* (MCAN/IR/07/Moheb-gh). After using the restriction enzyme, obtained fragments were subjected to electrophoresis in 2% agarose (Sigma-Aldrich, St. Louis, MO) at 80V in 1x TAE buffer, stained with safe stain (5 μL/100 mL), and visualized and photographed using a UV transilluminator.

### Confirming of Identification of Leishmania Species by kDNA-snPCR

A semi-nested PCR for detection of *Leishmania* spp. DNA was performed for amplification of variable area of the minicircle kDNA (with a slight modification) (22). The combination of primers LINR4 (forward), LIN17 (reverse) and LIN19 (reverse) were used in a semi-nested PCR technique. These primers were designed within the conserved area of the minicircle and contained conserved sequence blocks (CSB), CSB3, CSB2, and CSB1, respectively (23). The mixture was incubated at 94 °C for 5 min followed by 30 cycles, each consisting of 30 sec at 94 °C, 30 sec at 52 °C, and 1 min at 72 °C. After the last cycle, the extension was continued for a further 5 min. For the second amplification first PCR product was added to the PCR mixture with 1μM LIN19 primer for 33 cycles under the conditions as follows: 94 °C for the 30 sec, 58 °C for 30 sec and 72 °C for 1 min) and the final extension at 72 °C for 10 min.

Banding patterns of *L. tropica* and *L. major* were 760 and 560 bp, visualized on 2% aga-rose gel stained with safe stain.

### Sequencing and Phylogenetic Analyses

The PCR products from cutaneous *Leishmania* isolates (Table 1) were extracted from the gel using a Vivantis Gel Purification kit (Vivantis, Malaysia) according to the manufacturer’s protocols, were sequenced using the same forward and reverse primers used for amplification by an ABI 3730 sequencer (Bioneer, Daejeon, South Korea). The sequences were edited and manually checked with BioEdit Sequence Alignment Editor (24), aligned and compared with sequences from *Critidia fasciculate*, *Trypanosoma cruzi* by ClustalX 2.12 (25) (http://www.clustal.org/clustal2/). The similarities among our sequences were calculated (data not shown) and phylogenetic tree (Fig. 4) was constructed by Maximum Likelihood method in Tamura 3 parameter option for DNA sequences with a complete deletion procedure, by using MEGA6 software (Molecular Evolutionary Genetic Analysis Version 6) (26). The bootstrap scores were calculated for 1000 replicates.

### Statistical analysis

Descriptive statistics (median, range) were calculated for continuous variables using SPSS ver. 16 (Chicago, IL, USA).

### Ethical Approval

The patients were aware that their skin scrapings were needed for diagnosis of the disease using molecular diagnostic methods. The trial was reviewed and approved by the Ethics Committee of Tarbiat Modares University as well as the ethical committee of the Center for Diseases Control of Iran in accordance with the Helsinki Declaration and guidelines.

## Results

### Parasitological Results

All 648 slide samples and 3 references were checked for the presence of *Leishmania* by microscopy and all were positive. Average age was 20 yr (range, 7 months–78 yr), 55.3% males and 44.7% females. The time between the appearance of the lesions and presentation was 1–4 months for the cases infected with *L. major* and 3–9 months for cases infected with *L. tropica.* Two hundred-eleven patients (32.3%) presented the disease with multiple lesions, with a median number of lesions per patient of 1 (range, 1–5 lesions per patient). Hand, face, and feet were the most common sites of ulcers. In all lesions that were smear positive, average smear amastigotes density was grade 3 (1–10 amastigotes/10 high-power fields) (Table 2).

**Table 2:** The results concerning to patients characteristics

***Patients Characteristics***	***Results***
Average age	20 yr (range, 7 months –78 yr)
Sex	55.3% males - 44.7% females
Time between the appearance of the lesions and presentation	1–4 months (*L. major*)
	3–9 months (*L. tropica*)
Median number of lesions per patient	1 (range, 1–5 lesions per patient)
Average number of skin lesions	1.3 lesions per patient
The most common sites of ulcers	Hand, Face, Feet

## *Leishmania* Identification by kDNA-snPCR (LINR4, 17, 19) and PCR-RFLP Analysis / *(MO-TaqI)*

**Fig. 2:**
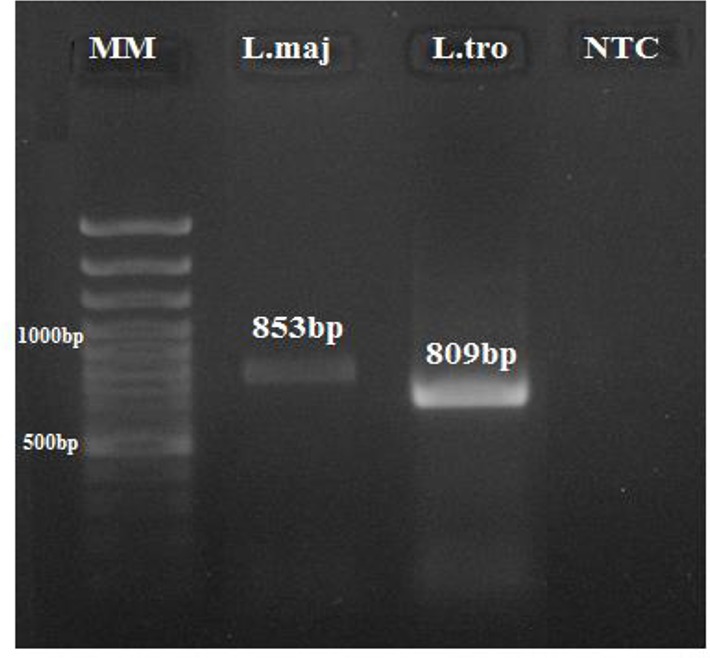
Agarose gel electrophoresis, showing PCR results by MO-ITS based primers on reference strains before enzymatic digestion. From left to right: Lane 1: MM: molecular weight marker. Lanes 2: *L. major* (853bp); lanes 3: *L. tropica* (809bp) and Lane 4: NTC, Non-Template Control

**Fig. 3:**
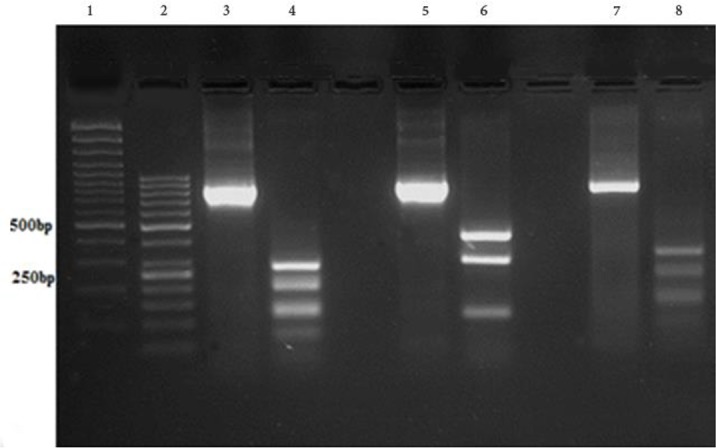
Agarose gel electrophoresis, showing PCR-RFLP results before (800–860bp) and after digestion with the restriction enzyme *Taq*I on reference strains. From left to right: Lanes 1 and 2 L: molecular weight markers (100, 50 bp). Lanes 3 and 4: *L. tropica*; lane 5 and 6: *L. major*, Lanes 7 and 8: *L. infantum*. After digestion by the restriction enzyme *Taq*I: *L. major*: 416, 296 and 141 bp.; *L. tropica*: 276, 193, 129, 115, 68 and 28 bp.; *L. infantum*: 326, 277, 142, 70 and 33 bp

The *Leishmania spp*. kDNA were detected by snPCR-LIN in all cases. Amplification of ITS-rDNA from all *Leishmania* isolates obtained from CL and 3 references were approximately 805–854 bp (Fig. 2). Digestion of amplicons with the *Taq*I enzyme produced banding patterns include the fragments of 416, 296 and 141 bp for *L. major* (853 bp), and fragments of 276, 193, 129, 115 and 68, 28 bp for *L. tropica* (809) and fragments of 326, 277, 142, 70 and 33 bp for *L. infantum* (848) (Fig. 3).

### Distribution of Cutaneous Leishmaniasis

**CL**: By comparison, to the patterns produced by the reference strains, 176 out of 648 isolates obtained from CL samples were identified as *L. tropica* and 478 as *L. major.*

**ZCL:** Dominant species in all over Iran is *L. major* from the center (Isfahan, Fars, Semnan, and Tehran), west (Ilam and Lorestan), southern (Fars), and southwest (Khuzestan) of the country.

**ACL:** *L. tropica* is distributed in the northeast (Khorasan-Razavi), north center (Tehran) of Iran and center of Iran in the city of Bam (Kerman Province).

### Sequencing, Similarities and Phylogenetic Tree

**Fig. 4:**
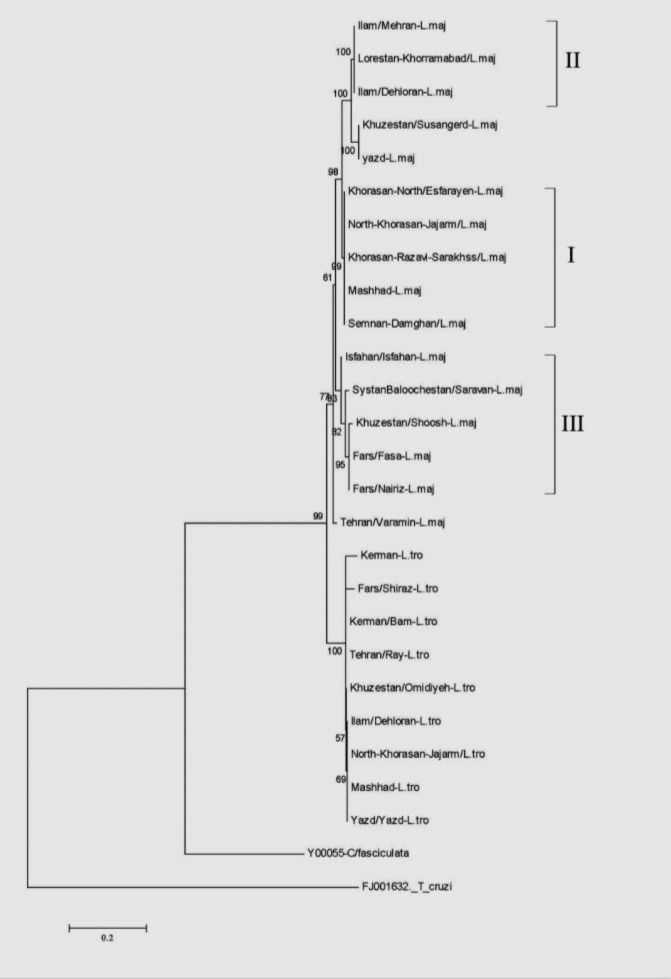
Phylogenetic tree of 25 Iranian *Leishmania* isolates from cutaneous cases of leishmaniasis and 2 isolates selected from GenBank, based on ITS-gene. The tree was constructed by using the Tamura3-parameter model in MEGA software version 6. The evolutionary history was inferred using the Maximum Likelihood method, supported by 1000 bootstrap replicates. The numbers above the branches indicate the percentage of bootstrap samplings percentages. Samples isolated in the present study were compared to isolates selected from GenBank^*^ ^*^ *T.cruzi/*FJ001632*/* and *C.fasciculata*/Y00055). I, II, III: Branches which intended to compare with distribution of rodent reservoirs in text

The *Leishmania* isolates were categorized into two main clads representing *L. major*, and *L. tropica*. The numbers above the branches indicate the percentage of bootstrap samplings. There was no clear grouping among the 25 isolates according to their geographical origin (Fig. 4).

Details of all specimens sequenced and 4 out of 25 sequenced amplicons submitted to GenBank are shown in Table 1. Phylogenetic trees using Maximum Likelihood showed intra-specific variations among *Leishmania* spp. isolates in this study and some other mentioned parasites extracted from GenBank. Analysis of ITS sequence in our samples showed the highest (100%) and lowest similarity (87%) (Data not shown).

## Discussion

Different climate conditions, environmental factors such as proper fauna and flora, common borders with countries which involve in war, poverty, poor sanitation, harboring refugees and in addition, lack of the effective vaccine, cause to rise in cases of diseases and eventually, crossing out the patients of their careers, high costs of treatments and finally, lead to economic losses; require to more accurate attention and survey on diseases in all over our country.

In Iran, many studies have been conducted to determine species of *Leishmania*, distribution of diseases and also evaluation of vectors and reservoirs fauna from different geographical areas. These studies included small sample sizes obtained from limited geographical regions (18, 27), as well as, the large scale of sampling in a broad geographic area (18).

Specific identification of *Leishmania* species usually depends on DNA amplification and sequence analysis. To detect *Leishmania* species in cases from the Iranian *leishmaniasis* foci, conventional and molecular methods employed by different molecular targets, NAGT gene (18) and nuclear ITS-rDNA (6, 27–29). Semi-nested PCR of kDNA, a highly sensitive technique of PCR has been used formerly for detection of Leishmania in sandflies (22, 30) and reservoirs (31), is used in the present study. Sequence of the ITS-rDNA gene was used for phylogenetic analysis of the Leishmania parasites in previous studies (32).

We used ITS-RFLP and ITS-sequencing approaches for the investigation of genetic diversity and population structure of two species of *Leishmania* from different endemic areas for CL in Iran. RFLP of ITS (18S rRNA–ITS1–5.8S rRNA–ITS2)-rDNA gene (*Taq*I enzyme) was used as the diagnostic and comparative methods for three *Leishmania* species (*L. tropica*, *L. major* and *L. infantum*) because of the size of the DNA fragments after the enzyme digestion and sequence of fragments before the enzyme digestion was used for constructing a phylogenetic tree.

Electrophoretic patterns of 648 CL isolates compared with reference strains showed that 478 (73%) and 176 (27%) isolates belonged to *L. major* and *L. tropica* species, respectively. The majority of *L. major* and *L. tropica* isolates were collected from rural areas, and urban areas, respectively. The dominant species in all over Iran is *L. major* in the center (Isfahan, Fars, Semnan, and Tehran), west (Ilam and Lorestan), south (Fars), and southwest (Khuzestan) of the country while *L. tropica* is distributed among the northeast (Khorasan-Razavi), north center (Tehran) and center of Iran in the city of Bam (Kerman Province). In terms of molecular epidemiology, our results are consonant with previous comprehensive epidemiologic study performed on patients and reservoirs (27).

In this study, there was a relationship between the incidence of disease with gender, sex and age. The incidence was higher in men than women and the median age of 20 yr old included patients that are inactive ages, work on farms or in open areas. The reason might be that more men work or sleep in the open areas and they are less covering than women are. Besides, they are more exposure to the infected sand flies.

Based on our phylogenetic tree, the *Leishmania* isolates were grouped into two main clads representing *L. major*, and *L. tropica*. Different patterns of *L. major* isolates obtained from various endemic areas of Iran and confirmed previous reports suggesting heterogeneity of *L. major* isolates analyzed by Single Strand Conformation Polymorphism (SSCP) and sequence analysis of ribosomal DNA (ITS) (32), (k)DNA-PCR by random amplified polymorphic DNA (RAPD) technique in Iran (33). In consonant, our phylogenetic tree shows levels of genetic heterogeneity between some isolates from different endemic areas as well as from the same endemic area. The heterogeneity seems between *L. major* isolates from different endemic areas as well as from the same endemic area (e.g. Khuzestan/Soosangerd/Shoosh). Moreover, also non-heterogeneous isolates belong to different endemic foci (e.g. Semnan/Damghan and Khorasan-Razavi/Sarakhss) as well as from the same endemic area (e.g. Ilam/Mehran/Dehloran).

Meanwhile, as expected in comparison with other studies that used smaller sizes of target fragments of selected genes for amplification to construct the phylogenetic tree, ITS-rDNA including 18s, ITS1, 5.8s and ITS2, chosen in the present study, is longer gene’s subject contains more different fragments and this matter will be followed by more variation as seen in our phylogenetic tree. These findings are in agreeing with other studies (18, 33) and are in contrast with a study (34), which reported *L. major* as the less divergent complex, and *L. tropica* as the more divergent complex. These different results may be due to variations in weather conditions, geographical regions, vectors, reservoirs or hosts and even in selected molecular targets in the studies. Since Iran has a wide geographic spread with various climatic conditions and also the presence of phlebotomine sandflies vectors in all parts of the country; the highest divergence among *L. major* isolates is acceptable.

Our results showed the slight similarities exist on *L. major* isolates from different parts of Iran (involved in sampling) and distribution of reservoir rodents which naturally infected with *L. major* in the same parts. According to our phylogenetic tree and in compare with previous studies; the northeast facing the center of Iran (include; north-Khorasan, Khorasan-Razavi and Semnan provinces, branch I) where *Rhombomys opimus* (35) and *M. libycus* are the dominant rodent species and among *L. major* isolates from the west to the center of Iran (include; Ilam, Khuzestan and Lorestan provinces, branch II) where *Tatera indica*, *Nesokia indica* and *M. libycus* are the dominant rodent species, in the center facing south and south of Iran (Isfahan and Fars, branch III) where *M. libycus* in the southwest is the dominant species (19) some similarities observed (36). To clarify the role of reservoirs in the epidemiology and genetic variation of *L. major* parasites in Iran, further studies with sampling of patients, vectors and reservoirs at the same time are needed. As seen in phylogenetic tree, variations in the sequences of the samples taken from the southern provinces (Fars) are more common where all three species are common there. Therefore, this molecular target could be considered adequate for the detection of discrimination of Iranian *Leishmania* isolates and gives us this opportunity to detect, identify, and construct the Phylogenetic relationship of Iranian isolates.

Wide range of human sampling from different provinces and various geographical and cultural conditions was the main limitation of this study.

## Conclusion

Iran has all the necessary conditions for the emergence of the disease in terms of environmental factors, vectors and host and also the surveys show the increasing rate of disease in recent years, so in order to have a real view of different aspects of disease, such extensive studies should be carried on. Despite the fact that both species are prevalent in Iran, but the dominant species is *L. major*, so this fact shows the importance of reservoirs rodents. Discrimination of Iranian *Leishmania* isolates using ITS gene gives us this opportunity to detect, identify, and construct the Phylogenetic relationship of Iranian isolates.
